# Retention in Care among HIV-Infected Adults in Ethiopia, 2005– 2011: A Mixed-Methods Study

**DOI:** 10.1371/journal.pone.0156619

**Published:** 2016-06-07

**Authors:** Yordanos M. Tiruneh, Omar Galárraga, Becky Genberg, Ira B. Wilson

**Affiliations:** Department of Health Services, Policy and Practice, School of Public Health, Brown University, Providence, RI, USA; UCL Institute of Child Health, University College London, UNITED KINGDOM

## Abstract

**Background:**

Poor retention in HIV care challenges the success of antiretroviral therapy (ART). This study assessed how well patients stay in care and explored factors associated with retention in the context of an initial ART rollout in Sub-Saharan Africa.

**Methods:**

We conducted a mixed-methods study at a teaching hospital in Addis Ababa, Ethiopia. A cohort of 385 patients was followed for a median of 4.6 years from ART initiation to lost-to-follow-up (LTFU—missing appointments for more than three months after last scheduled visit or administrative censoring). We used Kaplan-Meier plots to describe LTFU over time and Cox-regression models to identify factors associated with being LTFU. We held six focus group discussions, each with 6–11 patients enrolled in care; we analyzed data inductively informed by grounded theory.

**Results:**

Patients in the cohort were predominantly female (64%) and the median age was 34 years. Thirty percent were LTFU by study’s end; the median time to LTFU was 1,675 days. Higher risk of LTFU was associated with baseline CD4 counts <100 and >200 cells/μL (HR = 1.62; 95% CI:1.03–2.55; and HR = 2.06; 95% CI:1.15–3.70, respectively), compared with patients with baseline CD4 counts of 100–200 cells/μL. Bedridden participants at ART initiation (HR = 2.05; 95% CIs [1.11–3.80]) and those with no or only primary education (HR = 1.50; 95% CIs [1.00–2.24]) were more likely to be LTFU. Our qualitative data revealed that fear of stigma, care dissatisfaction, use of holy water, and economic constraints discouraged retention in care. Social support and restored health and functional ability motivated retention.

**Conclusion:**

Complex socio-cultural, economic, and health-system factors inhibit optimum patient retention. Better tracking, enhanced social support, and regular adherence counseling addressing stigma and alternative healing options are needed. Intervention strategies aimed at changing clinic routines and improving patient–provider communication could address many of the identified barriers.

## Introduction

Antiretroviral therapy (ART) has transformed HIV into a manageable disease; its effectiveness in treating and preventing HIV has been very well established [1–3]. However, success with ART depends on how closely patients adhere to their care regimens. Poor adherence increases the risk of viral rebound, resistance development, disease progression, further transmission of infection, and mortality [2,4–6]. Those interested in the broadest possible success for ART have increasingly focused on long-term retention of patients in treatment programs, especially in resource-poor settings where ART is rapidly expanding and drug options for future treatment are limited.

In sub-Saharan Africa, adherence to ART initially equaled or surpassed what was observed in resource-rich settings [7], with about 35% of patients having left care at 36 months after beginning treatment. Although mortality contributed substantially to those believed to be lost-to-follow-up (LTFU) care, many patients simply no longer reported to treatment facilities or caregivers [8]. While high mortality in African settings is often associated with late presentation and delayed initiation of ART [9,10], factors associated with LTFU vary substantially from one setting to another. Structural and socio-cultural factors such as treatment program characteristics, poverty, family responsibilities, and social relations are often reported to be the major determinants of patient retention in care [11].

This study identifies factors affecting retention and measures their effects for a cohort of patients in Addis Ababa, Ethiopia to answer the following three questions. First, to what extent are patients who began HIV treatment after the rollout of ART continuing to engage in care over the course of the study? Second, what are the characteristics of patients who were not retained in care? Third, why did people fail to stay in care? This study examines data from the initial rollout of ART in the study setting and adopts a mixed-methods approach to inform current strategies for improving patient retention within a specific economic and socio-cultural context, thereby adding valuable data to the literature.

## Materials and Methods

### Study Design

A retrospective cohort study, conducted among HIV-seropositive adults enrolled in HIV care, was complemented by a series of focus group discussions (FGDs) with patients recruited from the same clinic.

### Setting

The study was conducted at the HIV clinic of a tertiary teaching hospital in Addis Ababa, Ethiopia between 2005 and 2011. The clinic offers HIV testing and counseling as well as pre- and post-ART follow-up services for inpatients and outpatients. ART has been freely available since 2005 for eligible patients in accordance with national guidelines [12]. Institutional Review Boards from Northwestern University, Addis Ababa University, and the National Science and Technology Commission of Ethiopia approved the study.

### Participants

**Fig 1:**
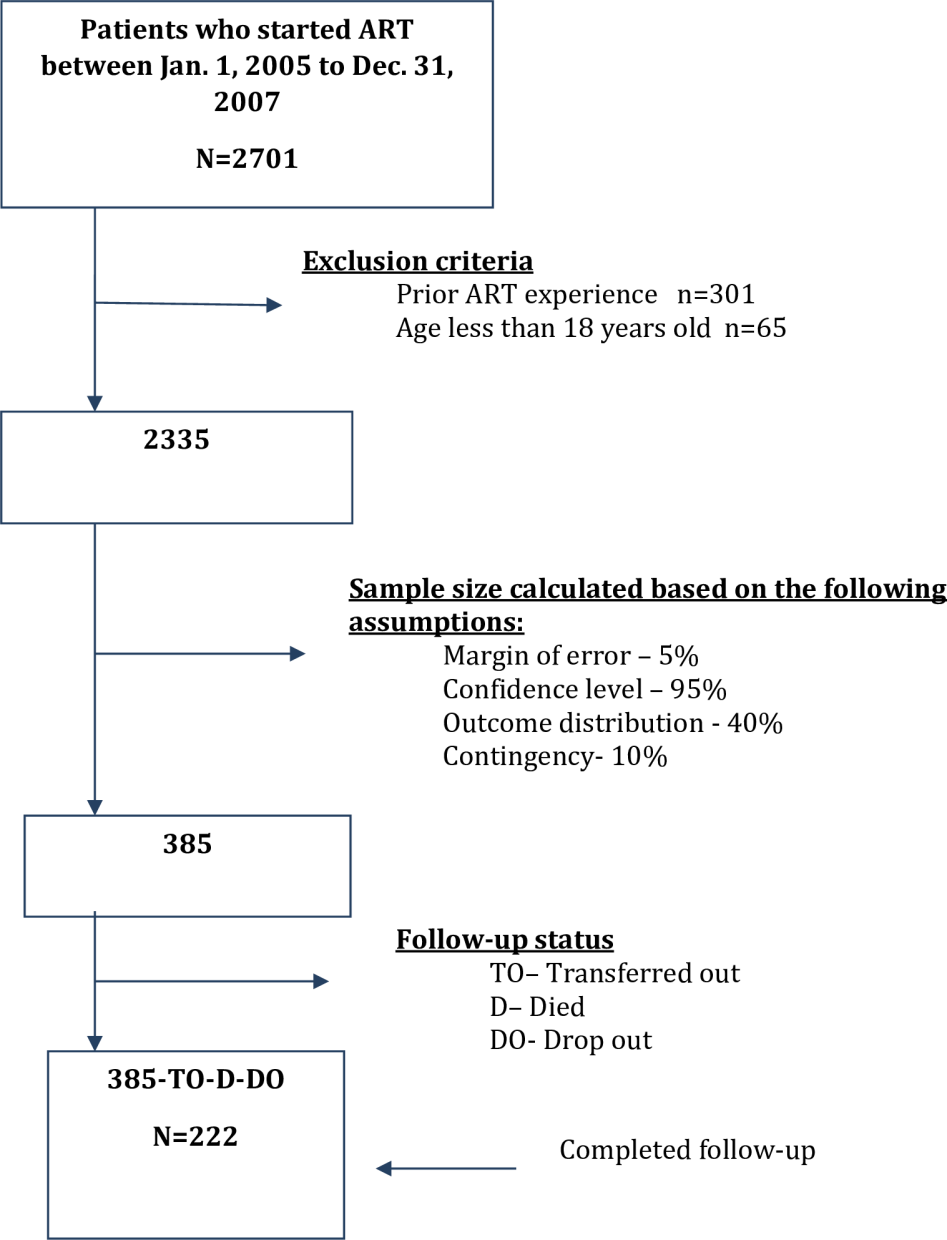
Flow chart of subject recruitment and follow-up.

A cohort of 385 patients was randomly selected from patients who started ART at the study clinic. To be included in the study, patients were required to have begun ART between January 1, 2005 and December 31, 2007 (the first two years after ART became freely available). Patients under 18 years of age and those who had started ART in another health facility were excluded from the study, as shown in Fig 1. The sample size was determined based on the following assumptions: 40% expected prevalence of becoming LTFU (estimated proportion), a 95% confidence level, a desired precision level of 5%, and a 10% contingency. From the eligible study population, subjects were selected using a random numbers table. Baseline participant characteristics including age, sex, religion, marital status, education, and employment status were compared with those of the broader patient population in a public HIV clinic setting in the study area.

### Quantitative Data

Data were abstracted from patient charts between June and September 2008 and participants were followed for an additional 3.5 years through December 31, 2011. Final follow-up status was determined based on the date of the last visit prior to the study’s end. Patient charts included standardized national intake and follow-up forms. Abstracted data from patient intake forms captured socio-demographic information, past medical history, general patient conditions, clinical illness stage, candidacy for ART and counseling, social conditions related to illness, and concerns of stigma (self-reported adherence concerns because of HIV stigma). Clinical data collected at ART initiation included CD4 count in cells/μL, bodyweight (kg), World Health Organization (WHO) clinical stage (assessed by healthcare providers per guidelines), ART regimen, and history of opportunistic illnesses including tuberculosis (TB). Functional status was categorized as working (able to perform usual work), ambulatory (able to perform daily living activities but not able to work), or bedridden (not able to perform daily living activities) based on patient reports. Data on the intake form were recorded by the healthcare provider for all patients at the first visit of the HIV chronic care clinic. On follow-up visits, ART regimen, treatment indicator tests (CD4 counts), regimen changes, reasons for regimen change, occurrences of pregnancy, and TB infection were abstracted.

### Quantitative Analysis

Our event of interest was becoming LTFU. We defined LTFU as having failed to appear for a scheduled clinic visit at least ninety days since the previous scheduled appointment. Patients whose deaths were confirmed, or who were transferred out, were censored. Patients who were alive and in care at the time of the database closure were administratively censored at their last visit date. Survival time was calculated in days from ART initiation to the date of the event (LTFU) or administrative censoring.

Data were analyzed using STATA 13 statistical software. Descriptive statistics were used to characterize the socio-demographic and clinical variables of the sample. Kaplan-Meier methods were used to depict and describe the occurrence of LTFU over time. Cox regression analysis was used to identify factors associated with LTFU and estimate hazard ratios adjusted for possible confounders. The Cox proportional hazard assumptions were checked using the Schoenfeld residuals method. Bivariate analysis was conducted for independent variables individually and variables that were significant at P ≤ 0.2 (with the exception of gender) were included in the multivariate model.

### Qualitative data

Six focus group discussions (FGDs) were conducted, three of which included only females, two of which included only males, and one of which included both females and males. All FGD participants were enrolled in HIV care and had been on ART for at least six months. FGD participants were selected randomly from the clinic when they came for their routine checkups or prescription refills. This means that they are a subset of the entire clinic population from which the cohort is sampled. Due to data privacy issues, we were unaware of the overlap of patients recruited for FGDs and those included in the cohort. Clinic nurses informed attendees of the opportunity to join an FGD. The first author, who facilitated the FGDs, explained the study in detail to willing participants, and informed them of anticipated meeting days and times. Between six and 11 participants attended each FGD and each lasted about an hour and forty minutes on average. Written informed consent was obtained from all FGD participants. To address confidentiality concerns, participants were advised to keep information from the discussion private and encouraged to use pseudonyms for the purpose of the discussion.

All FGDs were conducted in Amharic, the national language of Ethiopia, and were held at the study clinic in a private room. FGDs were digitally recorded with participants’ consent. Participants reflected on barriers to and facilitators of retention as well as reasons people become LTFU. In doing so they were reflecting on their own experience (past or intermittent) as well as experiences of others people they knew. Questions included: “What challenges do people face in attending clinic follow-up?” “Why do people disengage from care?” “From your experience or other people you may know, what makes staying in HIV care easy or difficult?” “What makes your illness management experience better or worse?” “How do you overcome challenges that hinder your ability to stay in care?” “Where and how do you get information about the benefits of and support for staying in care?” and “In your opinion, what do you think improves patients’ abilities to remain in care?”

### Qualitative analysis

FGDs were translated into English and coded using NVivo 10 software (www.qsrinternational.com) for analysis. Data analysis was informed by grounded theory and carried out inductively to construct categories of reasons affecting patient retention in HIV care [13]. Transcripts of FGDs were thoroughly read several times to identify reasons patients became LTFU. Texts that expressed similar thoughts/content were coded as provisional explanatory concepts. The first author performed all coding with several precautions to ensure rigor. After the initial coding, audio records, full transcripts, and notes were double-checked for coding accuracy to ground developing analyses. Codes appearing repeatedly under the same subject formed the basis for categories. Categories were reviewed to ensure that corresponding texts reflected their labels. Illustrative quotes for each category were retrieved from transcripts. Categories were interpreted for emerging patterns to identify similarities, differences, and interrelationships to integrate thematic explanations for retention [13,14]. Categories were grouped into barriers and facilitators that illustrated sociocultural and structural influences on retention.

Findings of quantitative and qualitative data were integrated using a contiguous staged narrative approach [15]. Quantitative findings are presented in the first half and qualitative results in the subsequent part of the results; themes are woven into the discussion. The integration resulted in expanding our understanding of challenges to retention as the FGDs provided explanations indicating why people failed to maintain engagement in care.

## Results

### Quantitative Findings

Table 1 presents the baseline socio-demographic and clinical variables pertaining to the 385 patients included in the cohort. Participants’ median age was 34 years (IQR: 28–40); 248 (64.4%) were women. The majority 303 (78.7%) were Orthodox Christians; 154 (40%) were married, 105 (27.3%) were never married, and 126 (32.7%) were divorced, separated or widowed. Close to half, 181 (47%), had elementary-level or no formal education and 121 (31.9%) were working, while 156 (41.2%) were unable to work or attend school due to illness at baseline. Over three-quarters of the patients, 326 (85.6%), began ART with CD4 counts of below 200 cells/μL (the mean CD4 count was 124 cells/μL (IQR: 61–173) and 283 (75.3%) were stage III or IV HIV. Only 55 patients (14.4%) had CD4 >200 cells/μL at ART initiation. We further categorized the group with CD4 < 200 into two groups based on the median value/50^th^ percentile for the group (101 cells/μL). A considerable number of patients, 160 (42%), had started ART with a baseline CD4 cell count of less than 100 cells/μL. About two-thirds (251) of the sample were on a Nevirapine-based regimen at ART initiation.

**Table 1:** Socio-demographic and Baseline Clinical Characteristics of Participants.

Characteristic	N(%)
Age (median (IQR) = 34 (28–40))	
18–34	201 (52.2)
35 and above	184 (47.8)
Gender (female)	248 (64.4)
Religion	
Orthodox Christian	303 (78.7)
Protestant Christian	33(9)
Muslim	44 (11.4)
Others*	5 (1)
Marital Status	
Never Married	105 (27.3)
Married	154 (40)
Divorced/Separated	54 (14)
Widow/Widower	72 (18.7)
Education	
No formal education	57 (14.8)
Primary	124 (32.2)
Secondary	137 (35.6)
Tertiary	63 (16.4)
Employment status	
Employed	121 (31.9)
Unemployed	102 (26.9)
Unable to work/study due to illness	156 (41.2)
Baseline CD4 count (mean = 124 cells/ μL)	
Under 100 cells/ μL	160 (42)
100–200 cells/ μL	166 (43.6)
Above 200 cells/ μL	55 (14.4)
WHO stage	
Stages I and II	93 (24.7)
Stage III	161 (42.8)
Stage IV	122 (32.4)
Baseline Functional Status	
Working	248 (66)
Ambulatory	89 (23.7)
Bedridden	39 (10.4)
ART regimen at initiation	
Nevirapine based regimen	251 (65.5)
Efavirenz based regimen	132 (34.5)
TB co-infection before ART	37 (9.6)
TB co-infection while on ART	11 (2.9)

* Others include Catholics and non-believers.

As indicated in Table 1, almost 10% (37) of the patients were co-infected with TB before the initiation of ART. Although the national guideline recommends that ART should be offered to all TB co-infected patients, in recognition of the possibility of high pill burden, drug toxicity, drug interactions, and Immune Reconstitution Inflammatory Syndrom (IRIS), the guideline prioritized initiating anti-TB treatment for co-infected patients [16]. Unfortunately, we do not have information indicating when anti-TB medication was started in relation to ART because the TB management was handled by TB clinics, not by the HIV chronic care clinic where we conducted our study. Over half 203(52.7%) of the patients were screened for TB while on ART and twelve (5.9%) developed active TB. As per the guideline, such patients started anti-TB medication along with ART. All of the twelve patients who developed TB while on ART were retained in care until the study’s end.

Eighteen (7.3%) of the women were pregnant and had received short-course antiretroviral medication to reduce the risk of mother-to-child transmission, consistent with national guidelines at the time of the study [17], before initiating long-term ART subsequently. Of these, seven (38.9%) were LTFU after the initiation of long-term ART while the remaining 11 (61.1%) stayed in care. Overall, five (2%) of the women in the study became pregnant while on ART and all of them stayed in care until the study’s end.

Follow-up time between ART start date and final status date (the last visit prior to December 31, 2011) ranged from 24 days to 2,296 days (mean = 1,306). At study’s end, 222 (57.7%) of the participants were retained, 10 (2.6%) were confirmed dead, and 39 (10.1%) were transferred out to other facilities. This left 114 (29.6%) who were LTFU for the remainder of the study period. The median time to LTFU was 1,675 days. Over half of those who were LTFU, 65 (57.7%), obtained this status during the first year after ART initiation.

**Fig 2:**
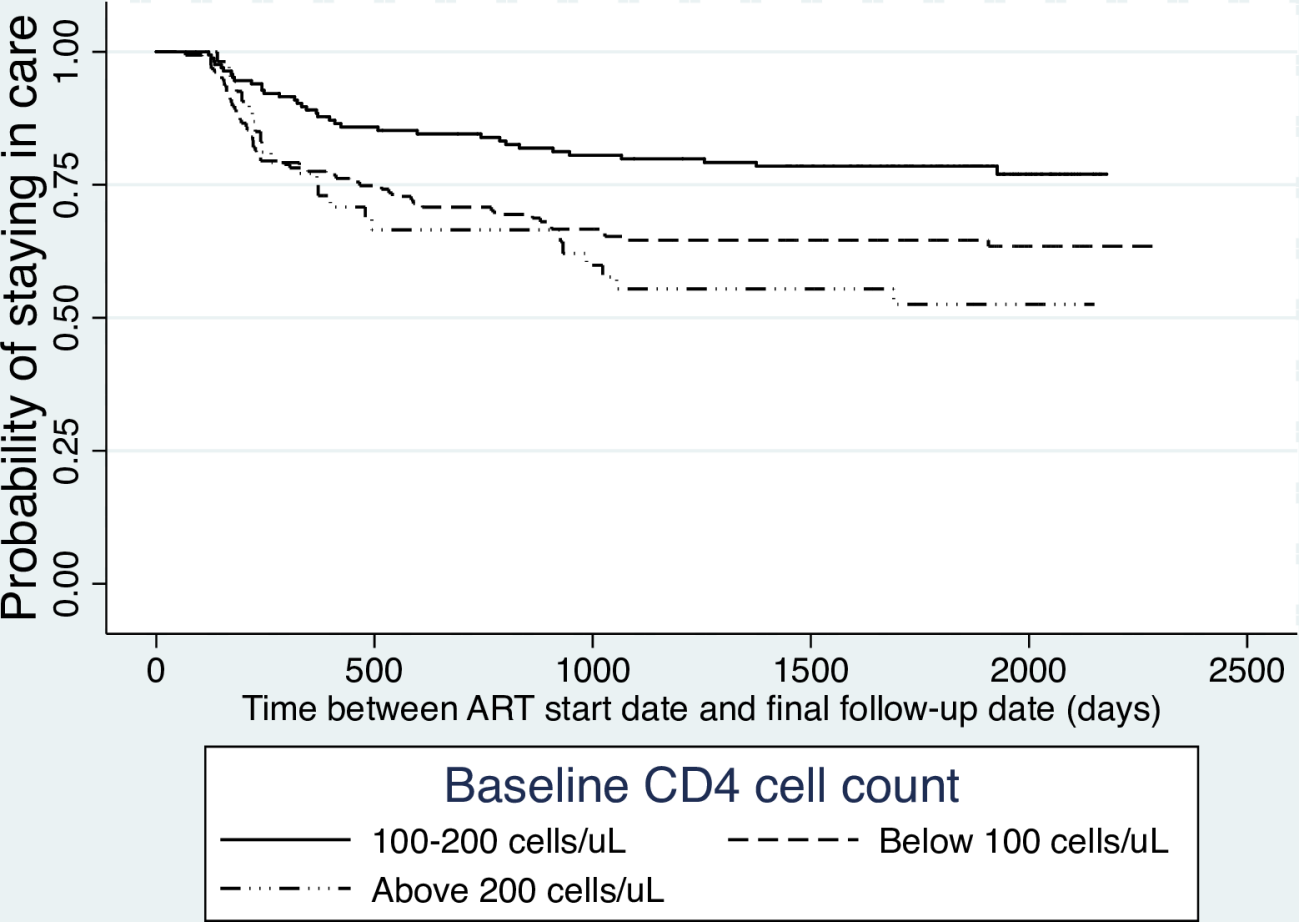
Kaplan-Meier LTFU Estimates by Baseline CD4 Cell Count of Participants, 2005–2011. The event of interest is LTFU—missing appointments for more than three months after the last scheduled visit or administrative censoring. People who died or transferred out their care to other facilities were censored.

Kaplan-Meier plots (Fig 2) show that, over a six-year period, the risk of being LTFU was substantially higher for people whose CD4 counts were <100 cells/μL and for those with CD4 counts of >200 cells/μL at baseline compared with those whose CD4 cell counts were between 100 and 200 cells/μL (p<0.05). There was also a statistically significant higher risk of being LTFU over time, comparing those with primary or no education to those with secondary education or above (P<0.05). Throughout the study interval, people who were bedridden at baseline were more likely to become LTFU compared with those who were working (P<0.01).

Similarly, in multivariate analysis, being LTFU was associated with baseline CD4 counts, educational level, and baseline functional status (Table 2). The final model also included gender, age, stigma concerns, and WHO clinical stage. Patients with CD4 counts <100 at baseline and those with CD4 counts >200 cells/μL at baseline had a higher risk of being LTFU (Table 2; HR = 1.62; 95% CI: 1.03–2.55; and HR = 2.06; 95% CI: 1.15–3.70, respectively; p<0.05) compared with those whose baseline CD4 counts were between 100 and 200 cells/μL. Bedridden participants at ART initiation were over two times more likely to be LTFU (HR = -2.1; 95% CI:1.1–3.8, P< = .05) when compared with people who were working. Having a lower level of education (HR = 1.5; 95% CI: 1.0–2.2, P = 0.05) was marginally associated with being LTFU when compared with those with secondary and above education.

**Table 2:** Univariate and Multivariate Cox Regression Analysis of Factors Associated with Lost-To-Follow-Up (LTFU)

	Univariate analysis		Multivariate analysis		
Characteristics	HR (CI-95%)	P-value	HR (CI-95%)	P-value	
Age					
18–34	1.33 (0.914–1.923)	0.137	1.34 (0.871–2.077)	0.181	
35 and above	1		1		
Gender					
Male	1.08 (0.740–1.578)	0.688	1.1 (0.700–1.723)	0.684	
Female	1		1		
Education					
Primary or no education	1.47 (1.008–2.132)	0.046*	1.5 (1.003–2.238)	0.048*	
Secondary or tertiary	1		1		
Stigma concern					
No	1.36 (0.937–1.969)	0.106	1.23 (0.820–1.857)	0.314	
Yes	1		1		
Baseline CD4 count					
Under 100 cells/ μL	1.85 (1.212–2.831)	0.004*	1.62 (1.028–2.550)	0.037*	
100–200 cells/ μL	1		1		
Above 200 cells/ μL	2.39 (1.410–4.044)	0.001*	2.06 (1.146–3.698)	0.016*	
WHO stage					
Stages I and II	1		1		
Stage III	1.46 (0.873–2.435)	0.150	1.23 (0.721–2.105)	0.445	
Stage IV	1.73 (1.020–2.934)	0.042*	1.17 (0.648–2.116)	0.600	
Baseline Functional Status					
Working	1		1		
Ambulatory	1.27 (0.810–2.000)	0.296	1.20 (0.742–1.951)	0.454	
Bedridden	2.92 (1.743–4.880)	0.000*	2.05 (1.105–3.804)	0.023*	

* P<0.05

### Qualitative Findings: Barriers to and Facilitators of Adherence to/Retention in Care

Four themes emerged from FGDs as factors that hindered optimal retention: stigma, dissatisfaction with care, economic constraints, and using holy water. Most of the emergent themes fall under broader social and structural factors related to the healthcare system and socio-economic realities of everyday life. Two themes emerged as facilitators: social support and restored functional ability, which were instrumental in helping participants prevent physical decline and mortality as well as stigma.

#### Stigma

Fear of HIV status disclosure was a considerable barrier to following clinic instructions and remaining in care. Participants across groups were discouraged by stigmatizing attitudes towards HIV. Some preferred to enroll in care facilities located relatively far from their neighborhoods, incurring additional costs in time and transportation. “They were going to transfer me to the clinic by my house, but I told [them] that I can’t go. It is [where] I grew up and got married. I can’t … let people point their fingers on my kids.”

Internalized feelings of HIV stigma were often found to hinder retention. “The difficulty is that, it [HIV] makes people to be inferior by far from other people … and you discriminate yourself.” Similarly, anticipated stigma was a significant barrier to retention especially for those engaged in domestic labor (employment decisions are at the discretion of individual employers) and for people who live in private rental residences: “if the landlord knows [the HIV status of the tenant] he might expel the person [with HIV] from the house.” Stigma was, however, a more frequently mentioned concern for female participants than for men. Male participants argued that stigma is a problem early in testing and seeking care as opposed to after initiating ART: “I do not see a close relationship between stigma and staying in care. Stigma plays a very pivotal role with getting tested and knowing one’s status …. After that, following therapy is a matter of deciding and remembering to take the medication.”

#### Dissatisfaction with care

Participants identified dissatisfaction with several components of the healthcare system as barriers to retention: the physical setting, the scarcity and cost of services for other illnesses, clinic routines and procedures, and provider factors. For example, location and the organization and delivery of HIV care were said to undermine privacy and were therefore inconvenient. Many participants felt stigmatized by the lack of a decent or private waiting area at the clinic: “The location is not right. I wear a big *netela* [traditional shawl] … to hide myself …. I want to pick it up [my medicine] in a safe zone where nobody would see me.” The difficulty of obtaining medication for opportunistic infections also meant additional costs for patients who struggle merely to subsist, as stated by the following participant:

The only medication available here [at the clinic] is for HIV. There is no medication [free] for other associated diseases when we are sick. We have to buy from outside [pay-based pharmacies], which is very hard [to afford]. You can’t take this meds [ART] when you are sick. I have encountered this difficulty many times.

Much dissatisfaction involved poor patient–provider relationships, due mainly to fragmentation of care. One-time or transient relationships with physicians prevent patients from establishing meaningful patient–provider relationships. Most participants characterized their visits as short, prescriptive, and impersonal and therefore they hesitate to raise concerns about their care with virtual strangers. Some participants reported that providers are overworked and thus fail to empathize with them, treat them nicely, or respond to their needs, often undermining patient–provider trust. A participant evinced this lack of trust as follows: “If I am seriously sick, I will not come here. Forgive me for saying this … I am not satisfied with how the physicians treat us here.”

#### Use of religious healing (holy water)

Many participants believe that holy water cures HIV: “I believe that HIV is God’s punishment. I do not think it is a virus; it is a devil and only God will cure it.” For others, being diagnosed with an incurable disease provides a rationale for seeking supernatural healing power: “We are sick and are affected by an incurable disease. We believe in the power of God that heals, but we appear to believe in that even more when we are near death.” Religious treatment challenges retention for people who perceive that spiritual care and medical treatment are mutually exclusive. This perception led some people, especially followers of Orthodox Christianity, to initiate ART late or discontinue treatment, as illustrated by the following quotes:

I said I would never take the medication …. I said God’s power that healed the woman who was bleeding for 12 years would heal me …. I stayed like that [without ART] for five years.

When people think that God will heal them, because they do not want to be healed by two things, they immediately stop the medication.

Participants reported that few HIV care providers discuss the possibility of safely integrating religious healing with conventional HIV treatment. Many patients do not even disclose their use of religious healing options to their providers because they do not feel comfortable doing so, they do not feel entitled to discuss such issues, or their providers never asked about the use of alternative healing options. However, on those rare occasions when patients had an opportunity to discuss it, disclosure was reported to increase retention: “I decided that I would not take the medication any more due to religious reasons … but the doctors and the nurses … convinced me to keep taking my medication.”

#### Economic constraints

Lacking food frequently emerged as a reason for poor retention. “People are forced to stop the medication [by] economic constraints,” said a participant referring to reasons associated with poor adherence and retention. Participants acknowledged the necessity of good nutrition despite the abject poverty in which many of them live. “There is … general thinking that the medication should not be taken without eating good food,” pointed out another participant, highlighting misconceptions of food requirements. Many participants who cannot eat regularly drop out of care, believing that medication without proper food is ineffective or even harmful: “My husband does labor … and for the past … month, he has been sick and never gone to work …. Taking these pills on an empty stomach is dangerous.” Such insights may also lead people to seek alternative healing options with less restrictive dietary constraints. A participant said: “Some of the people went to holy water … because they don’t have food to take the medicine with.”

#### Restored functional status

The most commonly mentioned factor motivating people to stay in care was improved health. Better health means needing less daily support and perhaps joining the labor force. This sense of independence was essential to achieving social rebirth, as caring for one’s family and children is an essential social expectation: “You will benefit everyone … if you take the medication without interrupting.” Such a sense of restored physical and social wellbeing helps many people see HIV as a manageable disease: “HIV is … tame … [and] not more severe than cancer.” Restored functional status also creates a sense of being fortunate to have access to treatment and has instilled hope that there will be a cure for HIV: “To the God who brought this [ART], nothing is impossible; He might bring the cure. What makes me sad is, to remember those people who passed away because they did not get access to treatment.

#### Social support

Social support was reported as instrumental in retention and medication adherence, as participants cited emotional, educational, material, and financial support from family, friends, non-governmental organizations, and care providers: “If your own people do not understand and support you, it makes you feel like stopping the medication and [dying] … when there are … people who could take care of you, you have a good reason to stay alive.” Social support is found to be helpful in fighting stigma, reducing the work involved in preventing inadvertent disclosures, establishing a community in which to share ideas, and obtaining resources.

## Discussion

This study generated three main findings. First, quantitative analysis revealed higher attrition rates among people in divergent disease stages—those with very low CD4 counts or those who had relatively higher CD4 counts at ART initiation. Second, over half of all LTFU cases (57%) occur during the first year of treatment and patients who were LTFU did not return to care (to the same facility). Third, sociocultural and structural factors were the most influential in determining retention of HIV patients in our study setting.

The LTFU rate observed in this study (30%) is comparable to rates found in other studies in low-income settings [18–21]. High attrition rates early in care have also been reported by many other studies [9,19,20], implying that interventions to improve retention should be emphasized during this critical time. Reluctance to return to care, at least to the same facility, could be attributed to shame and guilt for discontinuing treatment—which is considered a privilege—or fear of negative judgments for “defaulting” despite stern warnings at the beginning of treatment [22,23]. Healthcare providers should acknowledge the difficulty of staying in care and encourage reengagement no matter how long patients have avoided treatment. Acknowledging the inconveniences entailed by long-term treatment and normalizing non-adherence during counseling and follow-up visits would help encourage reengagement of people who may drop out of care for reasons that reflect our findings.

Our quantitative data revealed that those with CD4 cell counts of 100–200 cells/μL were more likely to stay in care than were people with lower or higher CD4 cell counts at baseline. Similar bimodal findings were reported in a study conducted in Nigeria [24]. The higher attrition rate among people with very low CD4 counts (<100) at ART initiation could simply mean they were too sick to pursue care or had died by study’s end [9,25]. Many studies have reported a similar trend [18,20,26,27]. On the other hand, several other studies have reported that the risk of being LTFU increases with relatively higher CD4 counts at baseline [28–31]. Perhaps patients who are not acutely ill see no urgency to remain in care. Alternatively, as indicated in our qualitative data suggesting that people tend to choose religious healing in hopes of finding a complete cure, less symptomatic people are stable enough to exhaust other healing alternatives [24,29,32–34]. This implies that late initiation of ART may not be the only reason for poor retention, as asymptomatic people may be reluctant to accept an HIV diagnosis or seek alternative healing options and therefore opt out of medical care [22,35,36]. Although current HIV guidelines raise the CD4 cell count threshold for initiating ART to 500 cells/μL [37] people with higher baseline CD4 counts who are relatively healthy at enrollment are equally at risk of poor retention [38]. However, the benefit of early ART initiation is undeniable as it increases the chances of positive treatment outcomes and reduces morbidity and hospitalization [6,39,40]. Additional efforts are needed to understand how to engage relatively healthy individuals in care, regardless of their perceived need for treatment or lack of physical symptoms.

Although concerns at the initiation of treatment that stigma might affect one’s adherence was not significantly associated with retention in care in our cohort, stigma was qualitatively identified as a major barrier to retention and multi-level intervention is needed to reduce it [32,41,42]. Interestingly, our female participants in the FGDs referenced stigma more frequently than our male participants, who argued that stigma is not a big concern once treatment is started. These differences could be explained by differential perceptions of stigma in men and women or by greater stigmatization of women living with HIV than of men. If this difference holds true in a larger sample, it has important implications for gender-specific stigma intervention, warranting further exploration of the relationship between stigma and gender, including differential effects of stigma at the various stages of the HIV care continuum.

Our qualitative findings echo others’ that unfavorable clinic experiences affect retention in care [32,43,44]. Routines and procedures at the study clinic seemed to affect retention if they induced concerns about stigma, as has been reported in other settings [45], suggesting that care providers could reduce LTFU with greater sensitivity to privacy and procedures that differentiate HIV patients from others [45]. Similarly, lack of continuity in care, non-responsive care, and inappropriate treatment discourage patients from remaining in care, as others have also reported [42,43,46,47]. Evidence suggests that patient-centered positive interactions promote engagement in care, improving treatment outcomes [48–50]. Therefore, interventions that improve patient–provider communication are highly recommended. Although the additional cost in time and money this might entail is often cited as a challenge [51], especially in resource-poor settings, we argue that efficient use of already-scarce resources depends on effective communication.

As reported by other studies in Ethiopia and several resource-poor settings [32,52–55], lack of food represents another barrier to retention in HIV care. The misconception that ART medications are harmful if taken without good food reduces retention. Amid abject poverty and a rising cost of living, however, social support from family, friends, patient organizations, and nongovernmental organizations provides vital care. Our findings confirmed the results of several other studies that social support is the most commonly identified facilitator for retention [47,50,56]. We recommend that communities support HIV care services by providing food subsidies and other incentives when appropriate, especially to knock down barriers to retention outside clinic settings.

Our FGDs revealed that many HIV sufferers prefer alternative treatments, mainly holy water, posing a common challenge to retention. Other studies in Ethiopia also found that using holy water was the most common reason for becoming LTFU [25,32,57]. When alternative treatment substitutes for ART, the choice is often informed by denial of HIV status [58], the perceived benefits and efficacy of the alternative treatment [52,59], lack of trust in healthcare providers [60], and side effects of medication [61]. Care providers should proactively assess the use of alternative therapies and discuss local beliefs, seeking safe integration of the two healing itineraries. Close collaboration with religious institutions might also be instrumental in promoting simultaneous use of ART and holy water [25], by convincing patients that religious and conventional HIV treatments are not mutually exclusive.

Most people who remain in care over the long run cite improved health with ART, enabling them to regain economic independence and reduce stigma [62]. Our study revealed that, although ART is free, treatments for other infections are unavailable or unaffordable, adding another level of difficulty to focusing on HIV care while suffering an acute illness that needs immediate attention. HIV care in resource-poor settings would benefit from expanding services for treating opportunistic infections as well as other non-HIV medical conditions.

Some of the factors we identified that discourage retention are structural and not easily actionable. However, we argue that many of them could be addressed by intervening at the health facility level. A study in Ethiopia revealed that facilities that sensitize healthcare teams to the need for retention, involve all stakeholders in the care continuum, ensure continuity of care, link care and support services with community organizations, shift tasks to multidisciplinary teams of adherence counselors and case managers, and implement better tracing activities generate higher retention rates [63]. Providers need to listen to their patients and assess their perceptions of how care is organized and delivered. Giving patients opportunities to ask questions and discuss their concerns promotes good patient–provider relationships, an important first step in assessing barriers to retention [22]. In light of the high volume of patients and overworked providers in resource-poor settings [64], offering extensive or elaborate discussions with patients may be difficult. However, designing service delivery models that respect patients’ basic rights and devising strategies to include meaningful communication could be instrumental in improving retention.

Our study is subject to several limitations. Since the quantitative data are based on patient charts that did not allow us to track patient status after being LTFU, we did not capture information on how many of the dropped cases were caused by death, which might explain the low death rate reported in our study, possibly introducing misclassification bias [65]. Similarly, we could not know how many patients self-referred to other facilities or how many patients who were transferred out to other facilities remain in care. We also relied on a single site and excluded treatment-experienced patients from our study so that we could record baseline information on all patients. This could possibly introduce selection bias due to higher early patient loss and mortality following initiation of ART in resource-limited settings, especially when ART programs were scaled up [66,67]. As a result, our findings may not be generalizable beyond treatment-naïve patients from a resource-limited urban setting. Although we have qualitatively explored potential reasons for poor adherence and retention, our FGD participants were clinic attendants who are therefore retained in care. Thus we did not capture reasons for poor retention given by people who are completely out of care. Notwithstanding these limitations, our dataset provides several benefits. One advantage is that we followed patients for a relatively long time from initial rollout. Our mixed-methods approach also enabled us to supplement our quantitative data with richer explanations from our qualitative data.

Retention is a critical determinant of optimal individual and public health outcomes and is likely enhanced by providing services that respond to patients’ medical, psychological, and social needs. Special attention to people with limited social support resources, stigma concerns, and concerns about treatment regimen and efficacy is warranted. Interventions designed to improve engagement in care should begin with ART initiation and continue throughout the course of treatment, particularly for those who are relatively healthy and might be reluctant to stay in care. Finally, community involvement is crucial for addressing social and economic barriers to retention. Strengthening social support mechanisms and mobilizing community resources could prove effective in improving engagement in care.
